# α/β Hydrolases: Toward Unraveling Entangled Classification

**DOI:** 10.1002/prot.26776

**Published:** 2024-12-02

**Authors:** Fatih Ozhelvaci, Kamil Steczkiewicz

**Affiliations:** ^1^ Institute of Biochemistry and Biophysics Polish Academy of Sciences Warszawa Poland

**Keywords:** α/β hydrolases, enzyme structure, homology modeling, MEROPS, peptidase, protease, protein family, S09, S33, serine protease

## Abstract

α/β Hydrolase‐like enzymes form a large and functionally diverse superfamily of proteins. Despite retaining a conserved structural core consisting of an eight‐stranded, central β‐sheet flanked with six α‐helices, they display a modular architecture allowing them to perform a variety of functions, like esterases, lipases, peptidases, epoxidases, lyases, and others. At the same time, many α/β hydrolase‐like families, even enzymatically distinct, share a high degree of sequence similarity. This imposes several problems for their annotation and classification, because available definitions of particular α/β hydrolase‐like families overlap significantly, so the unambiguous functional assignment of these superfamily members remains a challenging task. For instance, two large and important peptidase families, namely S9 and S33, blend with lipases, epoxidases, esterases, and other enzymes unrelated to proteolysis, which hinders automatic annotations in high‐throughput projects. With the use of thorough sequence and structure analyses, we newly annotate three protein families as α/β hydrolase‐like and revise current classifications of the realm of α/β hydrolase‐like superfamily. Based on manually curated structural superimpositions and multiple sequence and structure alignments, we comprehensively demonstrate structural conservation and diversity across the whole superfamily. Eventually, after detailed pairwise sequence similarity assessments, we develop a new clustering of the α/β hydrolases and provide a set of family profiles allowing for detailed, reliable, and automatic functional annotations of the superfamily members.

## 1 Introduction

**FIGURE 1:**
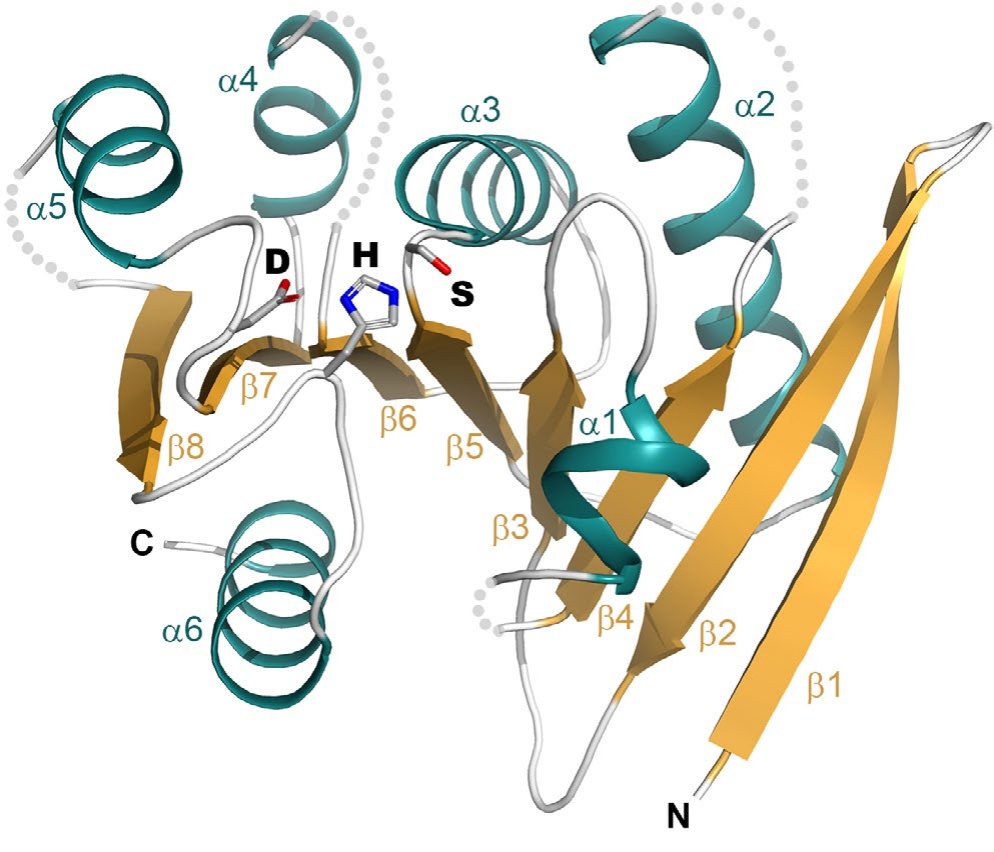
Structural core of α/β hydrolase‐like proteins on the example of wheat serine carboxypeptidase II from *Triticum aestivum* (pdb|3sc2 [9]). Only the core secondary structure elements are shown, for clarity. Catalytic triad residues are rendered with sticks.

Proteins classified as α/β hydrolases are found in a wide diversity of organisms representing all kingdoms of life, including viruses. They function as lipases, deacetylases, proteases, lactonases, epoxide hydrolases, peroxidases, lactamases, reductases, dehalogenases, lyases, or esterases of many substrates [1, 2]. Almost none of these functions is incidental within this superfamily and each appears within multiple separate protein families. We know many protein superfamilies displaying extreme sequence and structure diversity, for example, RNaseH‐like [3] or papain‐like [4], but they usually have some major, coherent functional theme like nuclease or peptidase. α/β hydrolases, even at their very definition by Ollis and collaborators in 1992 were being described as highly flexible regarding their catalytic potency [5]. The first structures commonly defining α/β hydrolases were lipase, dehalogenase, lactone hydrolase, peptidase, and acetylcholine esterase [5]. All of them retain a common structural core of eight‐stranded, twisted β‐sheet in 12435678 topology, flanked by six helices on both sides, as well as the spatially conserved catalytic triad of the nucleophile, acid, and histidine residues [5, 6, 7, 8] (Figure 1).

**FIGURE 2:**
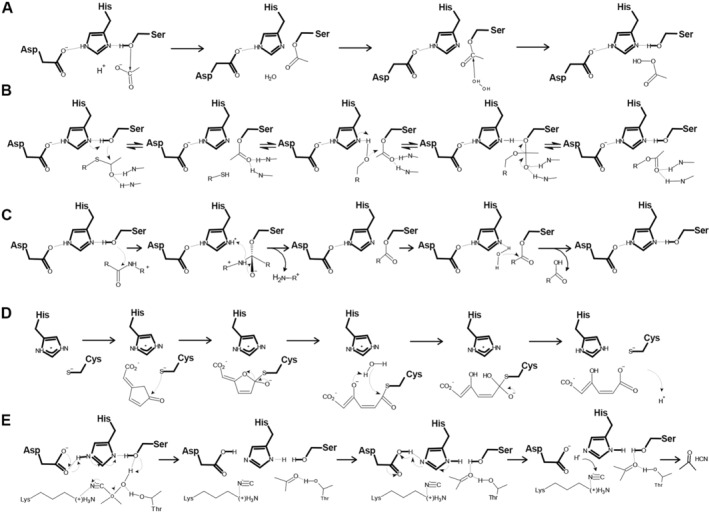
α/β hydrolase‐like enzymes are classified into multiple EC categories. (A) Chloroperoxidase, EC 1.11.1.10, after Hofmann et al. [14], (B) acetyltransferase, EC 2.3.1.175, adopted from Lejon et al. [15], (C) peptidase, EC 3.4.14.5, based on Roppongi et al. [16], (D) dienelactone hydrolase, EC 3.1.8.1, based on Cheah et al. [17], and (E) hydroxynitrile lyase, EC 4.2.1.37, adopted from Sharma et al. [18].

Although the structural core may differ in β‐sheet's curvature or helices alignment against the β‐sheet, it remains rather conserved and provides a firm basis for additional structural elements that ultimately shape the active site environment and substrate specificity [1, 10]. The catalytic site architecture is also conserved despite sequence and structure diversity. The nucleophile, mostly serine, is located at “nucleophile elbow,” a tight turn between strand β5 and helix α3; the acidic residue, usually aspartic or glutamic acid, follows strand β7; and totally conserved histidine localizes to a variable loop after strand β8. “Nucleophile elbow” also provides one of the oxyanion‐binding sites formed by the residue immediately following the catalytic nucleophile: either as the backbone nitrogen or residue sidechain [11], while the rest of the oxyanion hole comes from residues located usually after strand β3 [10]. As initially pointed out by Holmquist, the diversity of substrates of α/β hydrolases may be classified down to three general categories: (i) peptide‐, oxyester‐, and thioester‐bond, (ii) C—halogen or C—O bond, and (iii) C—C bond [2]. Based on subsequent fruitful 15 years of structural studies this classification was detailed by Rauwerdink and Kazlauskas into 17 different reaction mechanisms catalyzed by Ser‐His‐Asp triad alone [11]. Eventually, α/β hydrolases are classified into multiple enzymatic classes, differing even by the first EC number (Figure 2). Despite the function, the cleavage requires a nucleophilic attack by activated serine/cysteine (or even aspartate for C–halogen or C—O substrate) to form an enzyme–substrate intermediate, which is then cleaved by water [2, 12]. Only lyases catalyzing C—C bond breakage, despite having identical catalytic triad display different mechanisms of action: they skip acyl–enzyme intermediate, lack oxyanion hole but use serine as a donor of hydrogen bonds instead [13], or even do not require serine at all [11]. Nonetheless, the geometry of the catalytic site remains conserved so that with few mutations it is possible to switch between esterase and lyase, at least in certain α/β hydrolases [13].

The access to the active site's cavity as well as substrate specificity is regulated by additional structural elements inserted into the core fold, namely caps, lids, or flaps, as well as by supplementary protein domains covering the entrance [19]. For instance, lipases remain almost inactive with the catalytic site blocked by the lid when in the aqueous solution unless they reach the lipid phase which interacts with the hydrophobic surface of the lid, opens the catalytic cleft, and activates the enzyme [20, 21]. Active sites of proline exopeptidases on the other hand are covered by a seven‐bladed β‐propeller allowing specific substrates to pass through its central canal into the peptidase active site [22]. However, the majority of α/β hydrolase enzymes bind their substrates with smaller structural insertions, loops, or secondary structure elements extending their central β‐strands [1].

α/β Hydrolases are classified in multiple publicly available databases of differing scopes. PFAM, a general protein families database (ver. 35.0) [23], identifies 75 families within the AB_hydrolase clan (CL0028) covering over 2 million protein sequences. ESTHER database (ESTerases and alpha/beta‐Hydrolase Enzymes and Relatives) [24], provides a more detailed classification of α/β hydrolases at the level of their molecular function. α/β Hydrolases are represented also in the CAZy database of carbohydrate‐active enzymes [25]. The Lipase Engineering Database (LED) in turn was neatly designed to describe α/β hydrolase diversity based on their highly modular structure [19], and catalogs 200 000 proteins and almost 1600 Protein Data Bank (PDB) structures in a thoroughly curated manner. Representatives of α/β hydrolases are also included in MEROPS, a peptidase reference database that defines a curated hierarchy of clans and families of experimentally studied proteolytic enzymes [26]. Seven MEROPS families, namely S09, S10, S15, S28, S33, S37, and S82 belong to α/β hydrolases.

The very dense sequence space of α/β hydrolases and the ability of highly similar enzymes to catalyze different reactions (reviewed in [19]) impose several problems regarding their detailed classification. Substrate specificity of α/β hydrolases is shaped by structural add‐ons to the protein's catalytic core, sometimes extensive caps or big additional domains, but many times just loops or lids made of one or two helices [19]. Such minute traits are likely to disappear in statistical definitions for protein families (PFAM, ESTHER) so that multiple functional families of α/β hydrolases massively overlap. For instance, S09 and S33 families, even in MEROPS itself, despite peptidases containing thioesterases, lipases, methylesterases, carboxylesterases, epoxide hydrolases, and even hydroxynitrile lyases. In consequence, genuine peptidases are in the minority in these families so high throughput genome screening for these proteins would provide false positive results, which we experienced previously [27].

Although many previous studies already addressed the observed diversity of structures, catalytic mechanisms, and functions of α/β hydrolases, the problem of discerning between particular families and functions still remains open. The aim of this work is to identify new α/β hydrolase families, describe structural diversity within this superfamily, and propose supplementary family definitions that might help in reliable, automatic annotations. Using remote sequence homology detection methods we identify new PFAM families, PDB representatives (PDB90), and human proteins previously not classified as α/β hydrolases. Using sequence similarity networks between superfamily representative sequences we define clusters of functionally similar proteins and develop HMM profiles allowing for immediate and more detailed classification of any α/β hydrolase‐like protein. Based on manually curated structural comparisons we provide structure‐based multiple sequence alignment covering core structure elements for all defined clusters of proteins belonging to α/β hydrolase superfamily. Eventually, by combining sequence‐ and structure‐based analyses with additional data, like domain architectures and genomic contexts, we hypothesize on the potential functions of previously uncharacterized α/β hydrolase families.

## 2 Results

### 2.1 Identification of New α/β Hydrolases

Starting from the initial 75 PFAM families (classified to PFAM AB_hydrolase clan) and PDB90 representatives belonging to these families, with the use of sequence‐based remote homology detection method HHSEARCH we identified as α/β hydrolases additional 5 PFAM families which were not assigned to any superfamily. In the newest release of PFAM (ver.36.0) published during the preparation of this manuscript, two families: PF20591 (DUF6792) and PF10561 (C2orf69) became α/β hydrolases clan members, yet still no functional annotation is available for them. Three PFAM families previously classified to AB_hydrolase clan were discarded from our curated dataset: PF07167 (PhaC_N), which covers barely three initial β‐strands and overlaps with PF00561 (Abhydrolase_1); PF07176 (DUF1400)—domain preceding actual α/β hydrolase; and PF03893 (Lipase3_N) standing for a region N‐terminal to lipase domain. Eventually, the final dataset covers 77 PFAM families, 672 PDB90 structures, and 130 human proteins. Identified families span over 3.7 million proteins originating from all kingdoms of life.

### 2.2 Clustering of α/β Hydrolases

**FIGURE 3:**
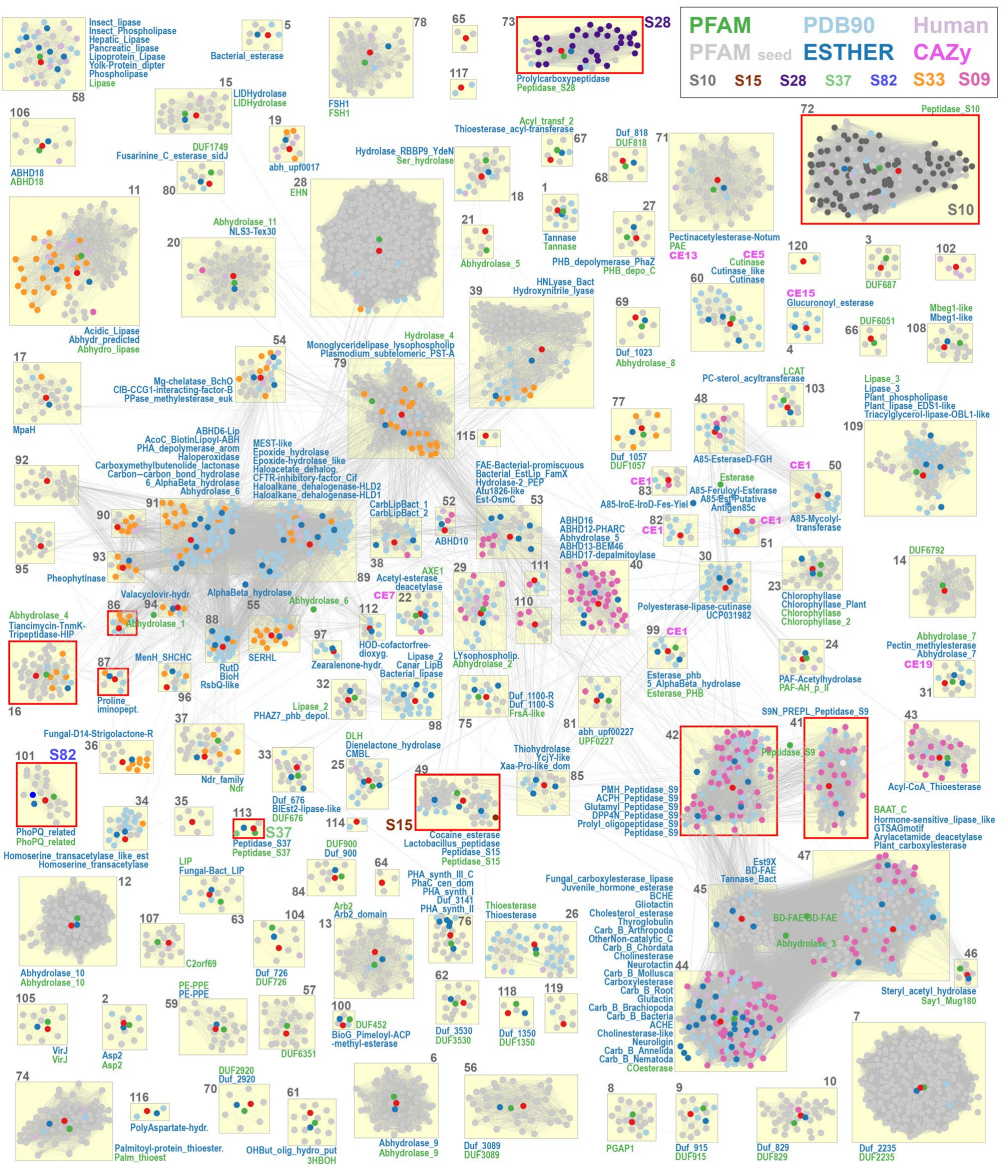
2D sequence similarity‐based clustering of α/β hydrolase‐like superfamily, including representatives of PFAM (gray) and MEROPS (multiple colors, see legend) families, PDB90 structures (light blue) and human proteome (pink). PFAM and ESTHER HMM profiles are marked with green and blue points, respectively. Lines connecting the points denote BLASTP mappings. Clusters are tagged with abbreviated PFAM, CAZy, and ESTHER family names written in green, pink, and blue font, respectively. Please notice the wide distribution of S09 and S33 peptidase families.

The easiest way to categorize identified sequences within a superfamily would be to map them into already existing families. However, due to overlaps between α/β hydrolase families, the usage of general HMM profiles would not allow for unambiguous assignments. Hence, based on pairwise sequence similarities evaluated with BLASTP scores we developed a supplementary clustering of α/β hydrolases sequence space covering representative sequences of all identified PFAM families (seed sequences), as well as MEROPS holotypes, PDB90, and human proteins. Eventually, α/β hydrolases were categorized into 120 clusters displaying structural and functional, with minor exceptions discussed below, consistency (Table S1 and Figure 3). One hundred and nine clusters contain at least one PFAM seed sequence, 80 include a PDB structure, and human proteins were present in 49 clusters. Two PFAM families are exceptionally spread across defined clusters: PF12697 (Abhydrolase_6) representatives may be found in 27 clusters, and PF00561 (Abhydrolase_1)—in 19; the next three families are present in five (PF00756, Esterase) and two (PF00326, Peptidase_S9; PF01674, Lipase_2) clusters. Out of seven MEROPS peptidase families S10, S15, S28, S37, and S82 localize within well‐separated single clusters, whereas S09 and S33 representatives are widely found in 19 and 22 clusters, respectively.

### 2.3 Development of HMM Profiles

For each cluster, we calculated the HMM profile and estimated family‐specific reliability cutoffs (encoded as “trusted cutoffs” in each profile). To test the performance of newly developed profiles we scanned 3 781 468 sequences of α/β hydrolase‐like proteins (collected using local PSI‐BLAST searches, see Section 4) with both original PFAM and newly developed HMM profiles. PFAM profiles identified α/β hydrolase domain in 3 487 071 protein sequences (92.2%) within the score trusted cutoff, and 3 753 025 (99.2%) if the score was not taken into account. However, 2 502 192 sequences (66.2%) were mapped into at least two PFAM families within trusted cutoff, and 1177 into 10 or more families. New, clustering‐based profiles identified 2 627 258 protein sequences (69.5%) within the defined trusted cutoff score (65 852 sequences, 1.7%, mapped to more than one cluster), and 3 765 265 (99.6%) without using the threshold.

There are regions in our clustering map (Figure 3) that still overlap, although not as massively as for original PFAM HMM profiles. The first region includes prolyl oligopeptidases (Cluster 41) and isopeptidases (Cluster 42) along with less overlapping feruloyl and acetyl xylan esterases (Cluster 45) and multifunctional Cluster 47 (vibralactone cyclase, heroin esterase, and hormone‐sensitive lipase). The second region covers Clusters 89 (epoxide hydrolase, dehalogenase, and oxidoreductase) and 91 (chloroperoxidase, lactamase, and hydrolase) with less overlapping Cluster 93 (chlorophyll dephytylase) and Cluster 39 (esterase/lyase).

### 2.4 New Clusters of α/β Hydrolases

Out of 120 clusters, 106 are represented in the PFAM AB_hydrolase clan, 17 of which are annotated as domains of unknown function (DUF). Ten clusters contain at least one PDB90 representative but no PFAM seeds, and one cluster consists of six human lipases only (DDHD1 phospholipase and its homologs). Eventually, three clusters cover protein families not included in the PFAM clan: PF19519 (DUF6051), PF05095 (DUF687), and PF09757 (Arb2). The latter family of histone deacetylases is structurally studied (pdb|5ikk_A [28]) and although it lacks a catalytic triad it retains structural features characteristic of this superfamily.

### 2.5 Uncharacterized Families

#### 2.5.1 DUF687 (PF05095)

Protein family of uncharacterized proteins found in *Chlamydia*. All family members lack catalytic residues and probably are either involved in substrate binding, or scaffolding within protein complexes. These proteins have a conserved additional C‐terminal bundle of six transmembrane helices similar to the domain in Abhydrolase_9 (PF10081) alleged lipases [29]. Hence, DUF786 members might function at the membrane as inactive homologs of lipids processing enzymes.

#### 2.5.2 DUF6051 (PF19519)

The bacterial family found predominantly in Bacteroidales, Chryseobacterium, Bacteroidetes, and other CFB group bacteria species. Family members retain catalytic triad as well as two structural insertions after strands β4 and β6. These additional elements interact with each other above the active site and form a double cap‐like structure charged positively from the active site cleft. Genes coding for DUF6051 proteins co‐occur with genes encoding 2TM and LytTR domains related to developing antigenic variation [30] and virulence regulation [31], respectively, as well as with long‐chain‐fatty‐acid‐CoA ligase, and PadR transcription regulator sensing environmental conditions [32]. Although *Chryseobacterium* species are rarely pathogenic, some species display potency to degrade demanding structures like cuticular exoskeleton [33]. Taking into account that many α/β hydrolase‐like families group lipases, DUF6051 proteins might function as enzymes cleaving specific membrane parts during the infection process.

#### 2.5.3 DUF6792 (PF20591)

The bacterial family is found in Firmicutes, mostly in *Bacillus*, *Priestia*, and *Alkalihalobacillus*. Members of this family have a small, mostly unstructured lid located after strand β4. The α/β hydrolase‐like domain is surrounded by two helical regions: N‐terminal comprising two long helices and another, four‐helix bundle, inserted in‐between strand β8 and catalytic histidine. None of these additional helices are predicted to be transmembrane and both display patches of negative charge on their surfaces, probably for interaction with other either proteins or macromolecules. Insertion of the whole domain (helical bundle, 229 residues) immediately preceding catalytic histidine, located on the loop, is unique in the whole α/β hydrolase‐like superfamily, and such arrangement might lead to catalytic site stabilization only upon substrate binding. Genes encoding DUF6792 representatives co‐occur with genes encoding PsbP‐like domains (PF18933); PsbP proteins are associated with photosystem II functioning and optimize the availability of Ca^2+^ and Cl^−^ ions in higher plants [34]. However, the more detailed function of DUF6792 proteins remains unknown.

#### 2.5.4 C2orf69 (PF10561)

Eukaryotic family of catalytically active α/β hydrolases found in animals. Human representative, C2orf69, is essential for mitochondrial respiratory chain functioning [35], however, no more details regarding its functions are known. This protein has a 29 residue‐long insertion after strand β4 modeled as rather unstructured, as well as an extensive unstructured region between helix αB and strand β5, unusually located on the opposite side of the protein than the catalytic pocket.

#### 2.5.5 DUF915 (PF06028)

Although structures of three representatives of DUF915 family have been solved (lin2722 products from *Listeria innocua* (pdb|3ds8), SE_1780 protein of unknown function from *Staphylococcus epidermidis* (pdb|3fle), putative cell surface hydrolase from *Lactobacillus plantarum* WCFS1 (pdb|3lp5)), their function remains unknown. Family members are present in Firmicutes, for example, in *Staphylococcus*, *Listeria*, *Streptococcus*, and *Enterococcus*. In *Listeria* genomes, DUF915 genes are surrounded by CynX/NimT family MFS cyanate transporter, FadR/GntR fatty acid‐responsive transcription factor which binds acyl‐coA [36], and AI‐2E family transporter of quorum‐sensing signal autoinducer 2 [37], whereas in Staphylococci it co‐occurs with AmaP (alkaline shock response membrane anchor protein), BCCT family transporter, zinc‐binding alcohol dehydrogenase, oxidoreductase, 6‐phospho‐β‐galactosidase, PTS transporter subunits EIIC and IIA. The genomic context might suggest functions related to cell membranes, but no more specific prediction might be made for now.

#### 2.5.6 DUF829 (PF05705)

Protein family widely present in Eukaryota. Human representative, TMEM53 (Q6P2H8) blocks cytoplasm–nucleus translocation of Smad proteins in osteoblasts [38] and has an inhibitory effect against SADS‐CoV by disrupting NSP8–NSP12 interaction for viral RNA synthesis [39]. It has a hydrophobic, two‐helix lid covering the catalytic site, suggesting that this protein becomes activated after reaching the lipid phase. The yeast genome encodes several DUF829 proteins; for instance, ICT1 protein (YLR099C) is an acyltransferase involved in membrane remodeling [40], LPX1 (YOR084W) is a peroxisomal matrix‐localized lipase [41], and LDH1 (YBR204C) is an esterase and triacylglycerol lipase for lipid homeostasis [42]. Therefore, besides the intriguing antiviral role of TMEM53, in general, DUF829 proteins might function as esterases/lipases at the cell's membranes.

#### 2.5.7 DUF3089 (PF11288)

This bacterial family is present mainly in α‐proteobacteria, CFB bacteria, γ‐proteobacteria, and others. In α‐proteobacteria DUF3089 genes are surrounded by Holliday junction resolvase (RuvX), holo‐(acyl‐carrier‐protein) synthase, and Asp‐tRNA(Asn)/Glu‐tRNA(Gln) amidotransferase subunits GatA, GatB and GatC. Modeled DUF3089 proteins from Paracoccaceae bacterium (A0A2D5TGJ5, α‐proteobacteria) and Bacteroidota bacterium (A0A3M1JDV7, CFB bacteria) have positively charged catalytic clefts, which suggests binding negatively charged substrates. Together with co‐occurrence with genes encoding nucleic acids processing enzymes, this might suggest that DUF3089 proteins are also engaged therein as acyltransferases.

#### 2.5.8 DUF3530 (PF12048)

Protein family found in *Pseudomonas* species. Due to the lack of a catalytic triad, family members probably play non‐enzymatic roles. Genomic neighborhood of DUF3530 genes is very conserved and includes LPS‐assembly protein LptD involved in the assembly of lipopolysaccharide at the surface of the outer membrane, phosphotransferase, *N*‐acetylmuramate alpha‐1‐phosphate uridylyltransferase MurU, TerB family tellurite resistance protein, sensor histidine kinase, response regulator transcription factor, and ABC transporter ATP binding protein. Modeled structure of DUF3530 family protein (Q9I5T9, PA0599) from *Pseudomonas aeruginosa* shows minimal α/β hydrolase domain with a negatively charged shallow cleft in place of the catalytic site. According to the STRING database, PA0599 protein co‐occurs with PA1463 (CheW chemotaxis protein), PA3353 (flagellar brake‐like protein), and PA5037 (ATPase) to list only a few. Besides the connection to the bacterial membrane, details about DUF3530 function remain elusive.

#### 2.5.9 DUF818 (PF05677)

Small protein family found exclusively in *Chlamydia* species. Their catalytic cleft is flanked with a small, two‐helix lid‐like structure inserted after strand β6, and three longer N‐terminal helices of which the second one shows a non‐zero probability of being transmembrane.

#### 2.5.10 DUF2920 (PF11144)

Protein family specific to *Campylobacter* species. In bacterial genomes, DUF2920 genes are flanked with genes encoding HAD‐superfamily hydrolase (haloacid dehalogenase‐like hydrolase), acyl carrier protein (transporting fatty acid chains between fatty acid synthases), pseudaminic acid cytidylyltransferase (catalyzing a step in the biosynthesis of pseudaminic acid used for flagellin modification), and UDP‐2,4‐diacetamido‐2,4,6‐trideoxy‐β‐l‐altropyranose hydrolase (also involved in pseudaminic acid synthesis). DUF2920 proteins have a small additional domain assembled from two insertions: first after strand β4 (inserted two β‐strands and two helices) and the second after strand β6 (two β‐strands), together forming a curved β‐sheet with peripheral α‐helices. This domain does not resemble other known protein structures to provide any hint about its functional implications.

#### 2.5.11 DUF1057 (PF06342)

Mostly eukaryotic family of proteins present in, for example, nematodes, mites, oomycetes, spiders, and gastropods. DUF1057 protein from *Caenorhabditis elegans* (O16919) are predicted to localize in mitochondria (DeepLoc: 0.78; WoLF PSORT: mito 17, cyto 15). Alphafold model of Hydrolase_4 domain‐containing protein (O16919) shows a three‐helix lid inserted after strand β6. However, we could find no hints for the potential function of these proteins.

#### 2.5.12 DUF900 (PF05990)

Bacterial family found in α‐proteobacteria, predominantly in *Rhizobium*, but also in *Sinorhizobium*, *Agrobacterium*, *Mesorhizobium*, and others. TMHMM identified an N‐terminal transmembrane helix in *Rhizobium meliloti* DUF900 protein (Q92YA4) and SignalP annotated it as lipoprotein signal peptide (Sec/SPII), although with low probability (0.44). DUF900 proteins have extensive, 11‐strand β‐sheet, and their catalytic site is buried between longer helix αB and an additional helix inserted after strand β7. Their function is unknown.

#### 2.5.13 DUF726 (PF05277)

A family of eukaryotic proteins, including human transmembrane and coiled‐coil domain‐containing protein 4 (TMCO4, Q5TGY1). Despite its name, TMCO4 does not seem to have any transmembrane element according to DeepTMHMM prediction, which is consistent with the 3D model of the protein. The majority of family members contain only one, α/β hydrolase‐like domain, with few exceptions: *Aspergillus* species have additional glutamyl‐tRNA amidotransferase complex subunit Gta3 (PF20978); *Alternaria* (major plant pathogens) have Pex14 N‐terminal domain (PF04695, peroxisomal membrane anchor binding PTS1 receptor); *Trichinella* parasitic roundworms have TRAM (PF01938), radical SAM (PF04055), and fucosyltransferase (PF17039 and PF00852, transfers fucose from GDP‐fucose to GlcNAc).

#### 2.5.14 DUF1350 (PF07082)

A family predominantly present in Cyanobacteria and eudicots, but also in monocots, algae, and plants. In Synechococcales, its representatives located in genomic proximity to acetate‐CoA ligase, solanesyl diphosphate synthase (ubiquinone synthesis [43]), HAD family phosphatase (housekeeping detoxification [44]), and peroxiredoxin (oxidative stress protection [45]), suggesting potential involvement of DUF1350 proteins in ROS stress maintenance in photosynthetic organisms. Yet, the detailed roles of these proteins remain to be uncovered.

#### 2.5.15 Cluster 64

A part of Abhydrolase_6 PFAM family (PF12697) found in high G + C Gram‐positive bacteria, mainly in *Streptomyces*, *Mycolicibacterium*, *Mycobacterium*, *Pseudonocardia*, and *Phodococcus* species. The detailed function of this family remains unknown, however, its members share a non‐canonical catalytic triad with serine residue substituted with aspartic acid, which makes them candidates for epoxide hydrolases. In the genomes of *Streptomyces*, genes coding for these proteins are surrounded by genes encoding NADPH‐dependent FMN reductase, LLM class flavin‐dependent oxidoreductase, NtaA/DmoA family FMN‐dependent monooxygenase, as well as multiple ABC transporter proteins: ATP‐binding, permease, substrate‐binding. Oxidoreductases and dehydrogenases, along with MFS transporters, are all encoded in genomic proximity in Mycolibacteria and Mycobacteria. This might suggest involvement in degradation pathways of indoles or styrenes which link the action of oxidoreductase with epoxidase [46].

### 2.6 Structural Diversity

**FIGURE 4:**
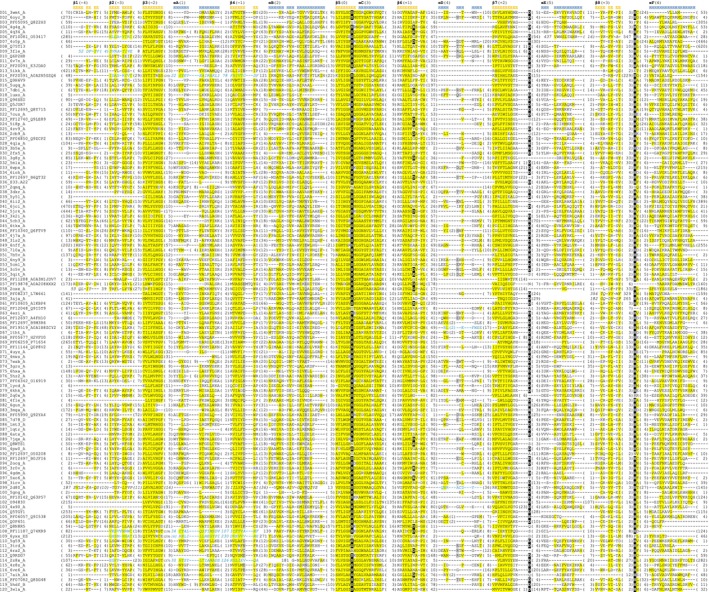
Structure‐guided multiple sequence alignment of structural core elements for α/β hydrolase representatives. Each sequence is tagged with cluster number and Uniprot/PDB identifier. The numbers provided in parentheses denote residues omitted from the alignment for clarity. Sequences written in italics indicate permutation and are preceded by the number of the first permuted residue, whereas sequences in turquoise are written backward and correspond to elements of reversed direction. Sequence conservation is marked with highlights following the scheme: Yellow—non‐polar, gray—charged, and black—confirmed or predicted active site residues.

In general, α/β hydrolases are conservative regarding their structural core. Central β‐sheet retains its characteristic twisted shape wrapping around the helices αA and αF across the whole superfamily. The most conserved are central hydrophobic β‐strands, especially strand 5 which together with helix αC form a hydrophilic elbow at the heart of the catalytic site. Peripheral elements like strands β1, β2, and β8 are more diverse; some enzymes lack strands β1 and β2 (lipase from *P. aeruginosa* (pdb|4o5p) [47] or human lysosomal phospholipase A2 [pdb|4x90] [48]) or strand 8 (ribosomal protein mL73 from *Leishmania major*, pdb|7aih [49]). In SE_1780 protein of unknown function from *S. epidermidis* (pdb|3fle) strands β1 and β2 are permuted and reversed in direction. Similar permutation and strand direction reversal may be observed in at least two lipases (extracellular lipase from *Pseudomonas* pdb|2z8x [50], lipase II from *Rhizopus niveus*, pdb|1lgy [51]), as well as in Mbeg1‐like (PF11187) and DUF6792 (PF20591) families. The most variable helix αD connects strands β6 and β7—in many structures it is either spatially displaced or deteriorated into a loop (see Figure 4).

### 2.7 Active Site Variation

**FIGURE 5:**
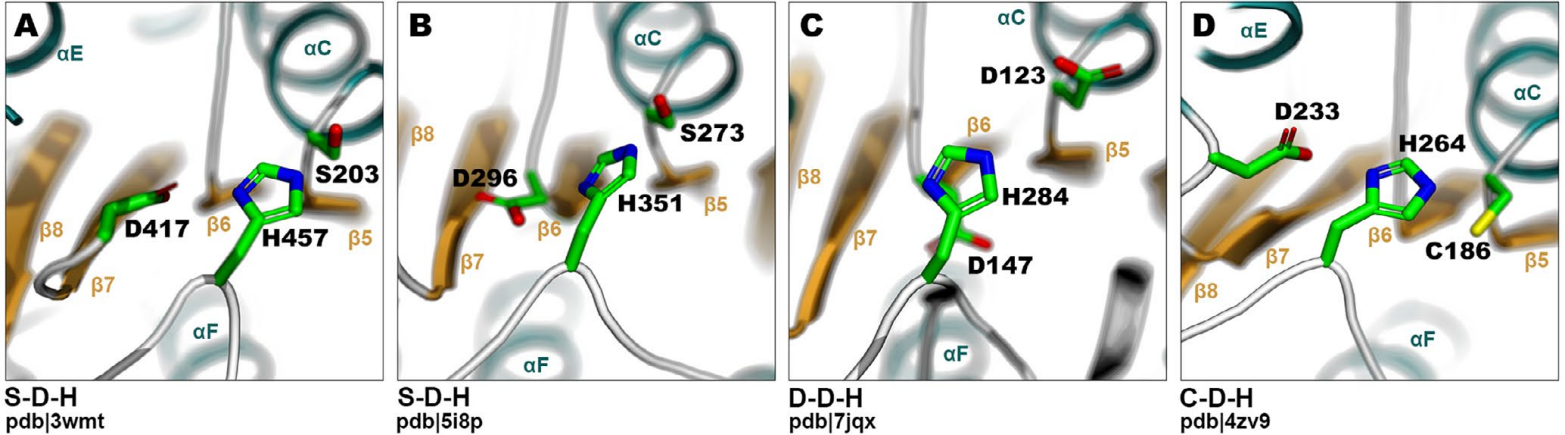
Examples of catalytic residues in α/β hydrolase‐like enzymes. (A) The most common triad in feruloyl esterase B from *Aspergillus oryzae* (pdb|3wmt [59]). (B) Example of acidic residue migration to strand β6 in human Platelet‐activating factor acetylhydrolase (pdb|5i8p [60]). (C) D‐D/E‐H triad is common in epoxide hydrolases, here in cif‐like epoxide hydrolase from *Burkholderia cenocepacia* (pdb|7jqx [53]). (D) *Escherichia coli* dienelactone hydrolase catalytic triad having serine substituted with cysteine (pdb|4zv9, no publication available).

Catalytic sites of α/β hydrolases are conserved regarding their spatial location within the structural core. Serine residue is always harbored at the nucleophilic elbow, and the histidine reaches the catalytic site from the loop connecting strand β8 and helix αF. Third, acidic residue in most cases is located at the C‐terminus of strand β7 (Figure 5A), but in many clusters it migrates to the strand β6 (Figure 5B). This reconfiguration is not specific to any particular structural or functional traits (see Figure 4 and Table S1). On the other hand, the composition of the catalytic triad may vary depending on the catalyzed reaction. The most common is the canonical S‐D/E‐H triad represented by the majority of clusters. Epoxide hydrolases change serine to aspartic acid residue resulting in D‐D/E‐H triad (clusters: 28, juvenile hormone epoxide hydrolase [52]; 89, epoxide hydrolase, pdb|7jqx [53], and 64 which is a part of Abhydrolase_6 family, Figure 5C). C‐D‐H triad, with cysteine instead of serine (Figure 5D), is present in dienelactone hydrolase, 4zi5 [54] (Cluster 25) but also in poly(3‐hydroxyalkanoate) depolymerase PhaZ, F8GXT6 [55] (Cluster 27), and polyhydroxyalkanoate (PHA) synthase, 5hz2 [56] (Cluster 76, DUF3141). Extraordinarily, some α/β hydrolases, like carboxy lyases, function with a D/E‐H dyad, without the hydrophilic serine (with some variations, discussed in [11]) (Cluster 39). Coenzyme A‐dependent lysophosphatidic acid acyltransferase, cgi‐58 (Q8WTS1, Cluster 90) also lacks catalytic serine and functions as lysophosphatidic acid acyltransferase [57], as well as a cofactor for the activity of lipases [58]. However, the exact catalytic site of this enzyme remains unknown.

### 2.8 Caps and Lids

**FIGURE 6:**
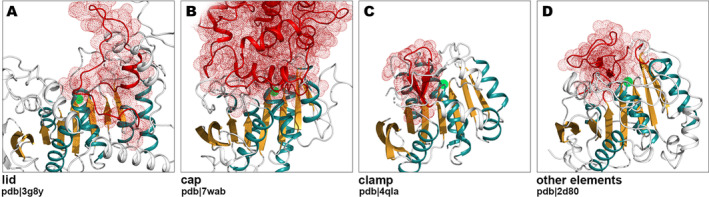
Common structural add‐ons to α/β hydrolase‐like fold core. (A) Lids, a small, mobile insertions managing the access to the catalytic site depending on conditions, putative hydrolase from *Phocaeicola vulgatus* (pdb|3g8y, no publication available). (B) Caps are much bigger and rigid structures covering active site cleft to allow only certain substrates to enter, prolyl endoprotease from *Aspergillus niger* (pdb|7wab [70]). (C) Clamps, the characteristic lid‐like structures of β‐hairpin, esterase domain from *Rhodothermus marinus* (pdb|5cml [69]). (D) Besides lids and caps, there is a diversity of other structural insertions lining the rim of the catalytic pocket, polyhydroxybutyrate depolymerase from *Penicillium funiculosum* (pdb|2d80 [71]). Discussed structural additions are rendered in red; the core α/β hydrolase‐like fold is colored in orange and teal; localizations of catalytic sites are marked with green dots.

The versatility of α/β hydrolases comes from their diversity in structural additions to the core, often referred to as lids or caps. Lids tend to be smaller and made of few helices at most but predominantly are mobile in managing access to the catalytic site depending on circumstances (Figure 6A). Caps in turn are bigger and immobile, and statically shape the access to the active site pocket which allows only specific substrates to enter (Figure 6B). Disregarding their size, the ultimate difference between lid and cap lies in their mechanics—for the lid two conformations should be observed, open, and closed [19]. Bauer et al. reported lids to be inserted into the structural core at five spots: β_+1_/β_+2_ (after β6 according to our numbering), β_−1_/β_0_ (after β4), β_−4_/β_−3_ (after β1), β_+3_/β_+4_ (after β8), and N_term_/β_−3_ (before β2). The most prevalent localization of the lid (over 40 clusters), is between strand β6 and helix αD. Its immediate proximity to the catalytic site couples substrate binding with the locking catalytic site in its active conformation. Consistently, the remaining observed lid insertion sites include: strand β8 (polyhydroxybutyrate hydrolase [pdb|5mtx] [61] and pancreatic lipase [pdb|2oxe] [62]), strand β4 (yeast FSH1 phospholipase [pdb|1ycd] [63, 64], bacterial esterase [pdb|1qlw] [65], and human phospholipase DDHD2 [O94830] [66], as well as within DUF6792 [PF20591] and Abhydrolase_8 [PF06259] [67] families), strand β2 (lipoprotein‐associated phospholipase A2, [pdb|5i8p] [60]), and strand β1 (Family I.3 lipase [pdb|2z8x] [50]). Ferulic acid esterases (pdb|7b5v, pdb|5cml) have a lid‐like structure, named clamp, consisting of a single β‐hairpin located after β‐strand 6 [68, 69] (Figure 6C).

Caps, similarly to lids, are often located after β‐strand 6, in clusters representing various functions, for example, lysosomal acid lipase (pdb|6v7n) [72], chlorophyllase (pdb|8fjd) [73], polyneuridine aldehyde esterase (pdb|2wfl) [74], hydroxynitrile lyase (pdb|1dwo) [75], methyl salicylate esterase (pdb|1xkl) [76], as well as in multiple peptidases: serine protease Hip‐1 (pdb|5ugq) [77], protease cathepsin A (pdb|4mws) [78], prolyl endoprotease (pdb|7wab) [70], tricorn‐interacting aminopeptidase F1 (pdb|1mt3) [79], prolyl aminopeptidase (pdb|1qtr) [80], secreted tripeptidyl aminopeptidase, Q9RDG7, and PhoPQ‐activated pathogenicity‐related proteins [81]. Additionally, juvenile hormone epoxide hydrolase (pdb|4qla) [52], hormone‐sensitive lipase (pdb|4j7a) [82], and DUF6051 (PF19519) have double caps—two structural insertions placed above catalytic site one on top of the other. However, the distinction between cap and non‐cap insertion may be approximate, because many α/β hydrolases, although devoid of lid/cap, still have core extensions shaping entrance to the active site, like PHB depolymerase (pdb|2d80) [71] with a crevice formed at enzyme's surface allowing single polymer molecule to enter catalytic site (Figure 6D).

### 2.9 Additional Domains

Representatives of many α/β hydrolase clusters possess family‐conserved additional protein domains, corresponding to their biological functions. For instance, fungal glucoronyl esterases (part of Cluster 4), found mainly in Basidiomycota and Ascomycota, have, among the others, additional fungal cellulose binding domain (PF00734, CBM_1). Abhydrolase_9 family members (PF10081, Cluster 6) are often fused with Abhydrolase_9_N domain (PF15420), which is a transmembrane domain found in lipases [29]. Similarly, poly‐β‐hydroxybutyrate polymerases (DUF3141, PF11339, Cluster 76), in many cases, have additional N‐terminal addon—PhaC_N helical domain (PF07176); thioesterases (PF08840, Cluster 43) tend to be preceded by acyl‐CoA thioester hydrolase/BAAT N‐terminal region (PF04775, Bile_Hydr_Trans) of β‐sandwich structure; histone deacetylases have N‐terminal catalytic domain (PF00850) followed by α/β hydrolase‐like inactive Arb2 domain (PF09757, Cluster 13) facilitating protein–protein interactions [83]. In bacterial thioesterases (Cluster 26, PF00975) α/β hydrolase can be preceded by several domains coherently functioning in peptide antibiotics biosynthesis: Condensation domain (PF00668), AMP‐binding (PF00501), AMP‐binding_C (PF13193), and additional phosphopantetheine attachment site domain (PF00550, PP‐binding). Finally, in eukaryotic phospholipases (e.g., phospholipase DDHD2, O94830, Cluster 102) α/β hydrolase domain is often fused to WWE (PF02825), SAM_1 (PF00536), and DDHD (PF02862) domains for interaction with other proteins.

Three peptidase families also have obligatory additional domains: prolyl oligopeptidases (PF00326, Cluster 41) require N‐terminal Peptidase_S9_N (PF02897) β‐propeller structure, isopeptidases (PF00326, Cluster 42) have N‐terminal WD40‐like Beta Propeller Repeat (PD40, PF07676), whereas bacterial dipeptidyl peptidases (PF02129, Cluster 49) have C‐terminal β‐sandwich domain (PepX_C, PF08530) and, in firmicutes, N‐terminal helical domain (PepX_N, PF09168) mediating dimerization.

### 2.10 α/β Hydrolase‐Like Peptidases

There are seven MEROPS peptidase families within the α/β hydrolase‐like superfamily, all belonging to the MEROPS SC clan: S09, S10, S15, S28, S33, S37, and S82. S10 family overlaps with the definition of PFAM PF00450 (Peptidase_S10 serine carboxypeptidase) and includes multiple eukaryotic carboxypeptidases: membrane peptidases (e.g., yeast's KEX1 processing toxin precursors and α‐factor [84], and facilitating cell fusion during mating [85]), vacuolar peptidases (e.g., carboxypeptidase Y [86]), or lysosomal protective peptidases (e.g., human CTSA [87]). S15 (PF02129, Peptidase_S15 X‐Pro dipeptidyl‐peptidase) is present in bacteria (Firmicutes, β‐proteobacteria, high G+C Gram‐positive bacteria, CFB bacteria) are peptidases removing N‐terminal dipeptides from their targets [88]. S15 includes also bacterial cocaine esterases enabling bacteria inhabiting coca plants to harness cocaine as a carbon source [89]. S28 (PF05577, Peptidase_S28 serine carboxypeptidase S28) is mostly fungal (Ascomycota, Basidiomycota) and plant family, but its representatives are found also in animals, including humans. This family includes carboxypeptidases (e.g., human lysosomal Pro‐X carboxypeptidase PRCP) and proline‐specific dipeptidyl peptidases (e.g., dipeptidyl peptidase 2, DPP7 [90]) important for peptide hormone signaling [91]. In plants, S28 peptidases may regulate gametogenesis [91]. S37 (PF05576, Peptidase_S37 PS‐10 peptidase S37) can be found predominantly in *Streptomyces*, *Bacterioides*, *Prevotella* species; S37 representative from *Streptomyces mobaraensis* , ptp (prolyl tri/tetrapeptidyl aminopeptidase) takes part in the maturation of transglutaminase by cleaving away N‐terminal tetrapeptide [92]. S82 (PF10142, PhoPQ_related PhoPQ‐activated pathogenicity‐related protein) members are barely characterized. Its representatives are involved in virulence [81] and can inhibit cell proliferation in *Dictyostelium* [93]. These five peptidase families are well‐defined and clearly discernible from the remaining α/β hydrolase‐like clusters, which is not the case for S09 and S33.

MEROPS representatives of families S09 (PF00326, Peptidase_S9, and other PFAM families) and S33 (PF00561, Abhydrolase_1, and others) are found in 19 and 22 clusters, respectively. Of these, two S09 clusters and three S33 clusters contain genuine peptidases. Taking into account the number of protein sequences assigned to each cluster, S9 bona fide peptidases account for 34.7% of all sequence space covered by all 19 clusters (321 741/927 482), while for S33—14.2% (102 992/727 819).

The S09 family covers two discernible peptidase clusters. The first includes prolyl oligopeptidases cleaving a variety of peptides (like neuropeptides or peptide hormones) after proline residue (S9A MEROPS subfamily, e.g. S09.001; *Novosphingobium capsulatum* prolyl oligopeptidase, pdb|1yr2 [94]), oligopeptidases B (e.g., S09.010; bacterial oligopeptidase B, pdb|6tf5 [95]) but also peptide cyclases (e.g., S09.078; PCY1, pdb|5uzw [96]) catalyzing transamidation instead of peptide bond hydrolysis. The second S09 cluster contains among the others isopeptidases targeting lariat knotted peptides (e.g., S09.032; sphingopyxin I lasso peptide isopeptidase, pdb|5jrk [97]), dipeptidyl aminopeptidases degrading bioactive peptides (S9B subfamily, e.g., S09.003; dipeptidyl aminopeptidase IV, pdb|2ecf [98]), and aminoacyl peptidases cleaving acylated amino acids for clearing cytotoxic denaturated proteins (S9C subfamily, e.g., S09.004; pdb|5l8s [99]), glutamyl endopeptidases (S9D subfamily, e.g., S09.021).

S33 peptidases fall into three clusters. The first contains prolyl aminopeptidases removing N‐terminal proline from specific peptides (e.g., S33.001; pdb|1qtr [80]). The second includes tripeptidyl‐peptidases (e.g., S33.002), and Hip1 peptidase cleaving GroEL2 (pdb|5ugq [77]). Finally, the third S33 cluster groups proline iminopeptidases (pdb|3nwo [100]), aminopeptidase grinding short peptides coming from tricorn degradation down into single amino acids (pdb|1mt3 [79]). The remaining clusters containing S09 and S33 non‐peptidase members represent a diversity of functions, including epoxide hydrolases, haloalkane dehydrogenases, lipases, esterases, lyases, thioesterases, and others (see Table S1).

## 3 Discussion

α/β Hydrolase‐like proteins form one of the more extraordinary superfamilies. Its members, numbering over 3.7 million, share a well‐defined structural core organized around an extensive, eight‐stranded β‐sheet of characteristic twist conserved across all families, as well as a catalytic triad tethered at the same location within the structure. However, relatively high structure conservation goes along with a high diversity of functions and one dominant setting of the active site is capable of catalyzing different enzymatic reactions. Moreover, switching between distinct enzymatic activities may require as little as two amino acid substitutions [13]. This phenomenon may be explained by the modular character of these proteins [19], whose function depends on small changes of residues lining the active site pocket, and structural elements added to the core. In consequence, similarities within conserved parts of these proteins may mask smaller differences to blend functionally distinct enzymes into the same families. Hence, α/β hydrolases cover 77 PFAM families which are highly diverse in terms of mutual sequence similarities; families like Abhydrolase_6 (PF12697), Abhydrolase_1 (PF00561), and Hydrolase_4 (serine aminopeptidase S33, PF12146) not only contain multiple, functionally unrelated proteins but also overlap regarding sequences belonging to them. On the other hand, many α/β hydrolase families are evolutionarily distinct and separate from other superfamily representatives.

Considering all the above, the development of the curated set of families/clusters definitions exposing a more detailed structure of α/β hydrolase superfamily must not rely solely on the automatic construction of sequence profiles; rather it should include the assessment of pairwise sequence similarities between representative proteins to define collectively similar groups. Such an initial set of clusters may be further extended to cover as many superfamily members as possible, yet with special attention paid to minimizing the overlaps. HMM profiles developed in this project cover the majority of α/β hydrolase‐like protein sequences while maintaining distinction between the clusters. Although the superfamily coverage of newly provided profiles within the estimated, family‐specific score thresholds, is lower compared to PFAM profiles (69.5% vs. 92.2%), they are still able to identify 99.6% of collected α/β hydrolases sequences; in the latter case, additional inspection of mappings may be needed to decide on an eventual family membership. Although general HMM profiles cover more protein sequences within reliability thresholds, they are not conclusive about the family assignment for 66.2% of the superfamily members which simultaneously satisfy reliability thresholds for two or more profiles. It should not be excluded that the non‐covered realm of the superfamily, or at least some part of it, would form additional clusters, not represented within the initial set of PFAM, PDB90, MEROPS, and human protein sequences.

Despite careful picking of each cluster member and reliability threshold estimation, some clusters display minor overlaps adding up to 1.7% of the superfamily members. The overlaps were present mainly between Clusters 41 and 42 (both peptidases), and within the group of Clusters 89, 91 (both containing proteins of diverse functions), 93 (chlorophyll dephytylase), and 39 (esterases/lyases). Observed, the functional diversity of Clusters 89 and 91 could not be separated with the presented approach. However, many broadly defined families were successfully split into smaller, more functionally coherent clusters, with extreme cases of Abhydrolase_6 (PF12697) distributed within 27 clusters and Abhydrolase_1 (PF00561) whose members may be found in 19 clusters.

**FIGURE 7:**
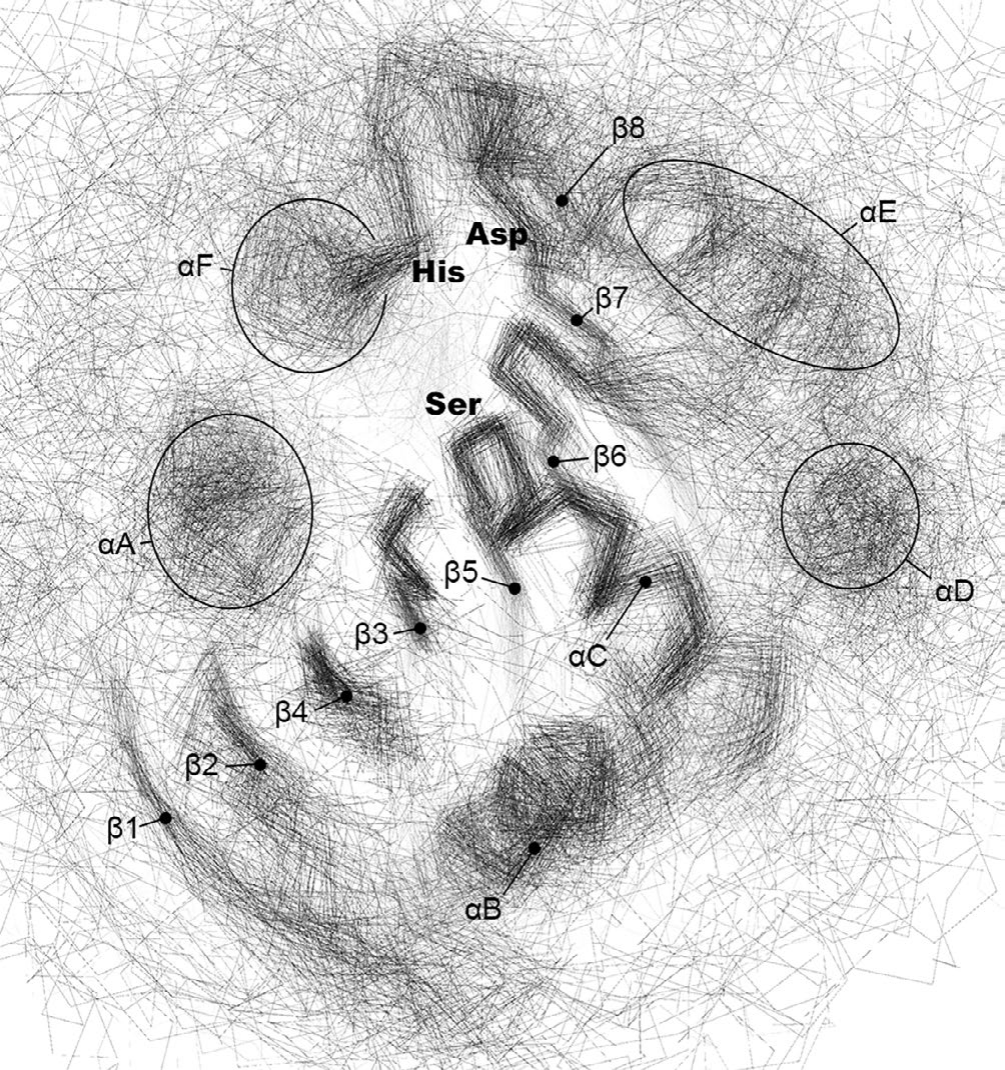
Superimposition of 3D structures representing all 120 defined clusters of α/β hydrolase‐like proteins. While strands β3, β4, β5, β6, β7, and helices αB and αC are well preserved, the remaining helices are much less pronounced and therefore seem blurred.

In spite of the conservation of the structural core (Figure 7) and catalytic residues signature, new α/β hydrolase families may still be hard to identify. This broad superfamily covers proteins from across the tree of life, diverged in amino acid sequences. Moreover, insertions of bigger elements to the structural core may disrupt multiple sequence alignments used for homology detection algorithms, hindering correct annotation. On the other hand, thanks to the contemporary advancements in computational methods, many families can now be classified as clans/superfamily members [23, 101]. Yet, such a rough assignment barely sheds light on the potential functions of so‐called “DUFs.” To hypothesize on their roles, one might need to take into account additional family traits, like domain architectures, genomic neighborhoods, or additional structural elements. However, functional prediction remains challenging for α/β hydrolases, because, as stated above, their detailed functions rely on small sequential or structural traits.

Additionally, despite the conserved structural core, the superimposition of PDB structures representing all superfamily members is not trivial in many cases. Although all structures retain nearly identical elements surrounding the hydrophilic elbow: hydrophobic β‐strands folding around helices αA and αF, β‐strands located at both rims of the central β‐sheet as well as helices may display greater distortions. Eventually, peripheral strands, like β1 and β2 may be missing, circularly permuted or even have reversed directions suggesting deeper evolutionary changes. The most structurally diverse element is helix αD connecting strands β6 and β7. The overwhelming majority of α/β hydrolases have a cap or lid inserted in‐between strand β6 and helix αD so that the latter functions as an elastic part of the hinge mechanism controlling the cap/lid functioning, explaining the extraordinary diversity of the helix.

Catalytic triad: Ser‐Asp/Glu‐His—one of the hallmarks of α/β hydrolases, although canonical in the majority of the clusters, may vary to some extent regarding catalyzed reaction and substrate processing. Hydrophilic serine is substituted with acidic residue in epoxide hydrolases, with cysteine in lactone hydrolases, or is missing at all in carboxy lyases. The acidic residue may migrate from strand β7 to immediately adjacent strand β6 while maintaining catalytic triad geometry. Nevertheless, all analyzed structures retain characteristic hydrophobic elbow (Figure 7) allowing for the immediate distinction between α/β hydrolases and other, similar protein folds, for example, Rossmann‐like with its crossover helix corresponding to the helix αB.

Superfamily‐wide structural comparisons provide a unique view of conserved and diverse features of α/β hydrolase‐like proteins. They also allow for more precise designation of protein domain borders, which further affects subsequent sequence‐based analyses. Based on pairwise sequence similarities between α/β hydrolase‐like domains in the initial set of representatives, using semi‐manual clustering we defined 120 protein clusters, with minor exceptions discussed above, coherent structurally and functionally. By developing HMM profiles for each cluster, we provide a ready‐to‐use database for further research projects requiring detailed annotation of α/β hydrolases.

## 4 Experimental Procedures

### 4.1 Preparing Initial Dataset

The initial set of previously known α/β hydrolase‐like proteins contained members of AB_hydrolase PFAM [23] clan (CL0028) and corresponding PDB90 representatives (PDB structures clustered to 90% of sequence identity to reduce redundancy), as well as peptidases from MEROPS database [26] and classified to the SC clan (families: S09, S10, S15, S28, S33, S37, and S82).

### 4.2 Identification of New α/β Hydrolase Superfamily Members

New members of the α/β superfamily were identified using the sequence‐based remote homology detection method HHSEARCH. HHSEARCH, a part of HHSUITE package [102], is a remote homology detection program which, instead of seeking for similarities between raw sequences, or even sequence profiles, boosts its sensitivity and specificity by comparing meta‐profiles—statistical objects encoding information about residue conservation enriched with predicted secondary structure patterns. HHSEARCH results were additionally verified according to the conservation of essential structural core elements and catalytic residues.

### 4.3 Clustering the Realm of α/β Hydrolases

Obtained set of protein sequences consisting of PFAM seed sequences, PDB90 and MEROPS representatives, and human proteins, was manually clustered based on pairwise sequence similarities (BLASTP [103], *E*‐value threshold 1 × 10^−4^) with the use of Cytoscape program [104], and AutoAnnotate Cytoscape app [105].

### 4.4 Preparing HMM Profiles

For each cluster, sequences of its members were aligned using MAFFT [106] (localpair, maxiterate 1000). Based on multiple sequence alignments, HMM profiles were built using HMMBUILD [107]. Since the initial set of HMM profiles was developed based on a very limited count of sequences which might affect their performance, we extended our analysis to the whole superfamily. For that, we collected all superfamily members using PSI‐BLAST [108] runs against the nr non‐redundant database (downloaded on Feb 24th, 2024) with one representative sequence per cluster as a query. The resulting dataset of 3 781 468 unique sequences was scanned with newly developed HMM profiles using HMMSCAN [107]. Based on the resulting bitscore distributions, for each profile, we selected a new set of protein sequences, clustered them with CD‐HIT [109] to 90% sequence identity, and repeated the procedure of profile calculation to include a wider set of homologs. This step differed from the initial HMM creation procedure in multiple sequence alignment options—instead of an exhaustive localpair iterative approach we used “—auto” flag to MAFFT to reduce computation time for bigger alignments. Eventually, the final scanning of 3 781 468 superfamily members with the updated HMM profiles allowed us to estimate reliability thresholds that balance profile's sensitivity and selectivity.

### 4.5 Additional Analyses

Domain architecture analyses were based on HMMSCAN searches against the PFAM 35.0 database. Cluster mappings to the CAZy database were performed using HMMSCAN against our newly developed HMM database with CAZy representative sequences used as a query. The genomic neighborhood was derived from the NCBI genomic data with the use of the E‐UTILS package. Taxonomic distributions were obtained from the NCBI taxonomy database provided along with the nr sequence database. Transmembrane elements detection was made with DeepTMHMM [110]. 3D structures for proteins representing clusters without known PDB structure were modeled using AlphaFold2 [111]. All structures were superimposed with the use of SPDBV [112], and manually curated multiple structure alignment was prepared in Discovery Studio Visualizer. Alignment manipulations/preparations were made in SEAVIEW [113]. All structures were visualized using the PYMOL program (https://pymol.org).

## Author Contributions

**Fatih Ozhelvaci:** investigation, writing – original draft, formal analysis. **Kamil Steczkiewicz:** conceptualization, investigation, funding acquisition, writing – original draft, methodology, validation, visualization, writing – review and editing, software, formal analysis, project administration, supervision, resources, data curation.

## Conflicts of Interest

The authors declare no conflicts of interest.

## Supporting information


**Table S1.** Summary of α/β hydrolase clusters defined in this study. Columns: no.—cluster number; PFAM—PFAM families of which representative sequences are present in the cluster; PDB90—PDB representative structures; MEROPS—MEROPS family members; HUMAN—human proteins (Uniprot accession codes); catalytic residues—catalytic triad residues with the additional distinction between acidic residue located after strand β6 or β7 (if the residue could be found within any arrangement of the catalytic triad in proteins from the cluster, it is marked in the respective column; hence, the presented residues denote a sum of triad combinations within the cluster, and particular representatives may have fewer catalytic residues); function—brief functional characteristic of the cluster; and taxonomic distribution for cluster members.

## Data Availability

Additional data, including HMM profiles and superimposed protein structures are available through Zenodo repository under DOI 10.5281/zenodo.11182431.
